# Evaluation of coagulation activation after Rhinovirus infection in patients with asthma and healthy control subjects: an observational study

**DOI:** 10.1186/1465-9921-15-14

**Published:** 2014-02-07

**Authors:** Christof J Majoor, Marianne A van de Pol, Pieter Willem Kamphuisen, Joost CM Meijers, Richard Molenkamp, Katja C Wolthers, Tom van der Poll, Rienk Nieuwland, Sebastian L Johnston, Peter J Sterk, Elisabeth HD Bel, Rene Lutter, Koenraad F van der Sluijs

**Affiliations:** 1 Department of Respiratory Medicine, Academic Medical Centre, Meibergdreef 9, 1105 AZ Amsterdam, The Netherlands; 2 Experimental Immunology, Academic Medical Centre, Meibergdreef 9, 1105 AZ Amsterdam, The Netherlands; 3 Experimental Vascular Medicine, Academic Medical Centre, Meibergdreef 9, 1105 AZ Amsterdam, The Netherlands; 4 Department of Medical Microbiology, Academic Medical Centre, Meibergdreef 9, 1105 AZ Amsterdam, The Netherlands; 5 Center of Experimental and Molecular Medicine, Academic Medical Centre, Meibergdreef 9, 1105 AZ Amsterdam, The Netherlands; 6 Experimental Clinical Chemistry, Academic Medical Centre, Meibergdreef 9, 1105 AZ Amsterdam, The Netherlands; 7 Laboratory of Experimental Intensive Care and Anesthesiology, Academic Medical Centre, Meibergdreef 9, 1105 AZ Amsterdam, The Netherlands; 8 Department of Vascular Medicine, University Medical Centre Groningen, Hanzeplein 1, 9700 HB Groningen, The Netherlands; 9 Airway Disease Infection Section, National Heart and Lung Institute, Imperial College London, Norfolk Place, London W2 1PG, UK

**Keywords:** Rhinovirus, Coagulation, Fibrinolysis, Asthma, Microparticles, Inflammation

## Abstract

**Background:**

Asthma exacerbations are frequently triggered by rhinovirus infections. Both asthma and respiratory tract infection can activate haemostasis. Therefore we hypothesized that experimental rhinovirus-16 infection and asthmatic airway inflammation act in synergy on the haemostatic balance.

**Methods:**

28 patients (14 patients with mild allergic asthma and 14 healthy non-allergic controls) were infected with low-dose rhinovirus type 16. Venous plasma and bronchoalveolar lavage fluid (BAL fluid) were obtained before and 6 days after infection to evaluate markers of coagulation activation, thrombin-antithrombin complexes, von Willebrand factor, plasmin-antiplasmin complexes, plasminogen activator inhibitor type-1, endogenous thrombin potential and tissue factor-exposing microparticles by fibrin generation test, in plasma and/or BAL fluid. Data were analysed by nonparametric tests (Wilcoxon, Mann Whitney and Spearman correlation).

**Results:**

13 patients with mild asthma (6 females, 19-29 y) and 11 healthy controls (10 females, 19-31 y) had a documented Rhinovirus-16 infection. Rhinovirus-16 challenge resulted in a shortening of the fibrin generation test in BAL fluid of asthma patients (t = -1: 706 s vs. t = 6: 498 s; p = 0.02), but not of controls (t = -1: 693 s vs. t = 6: 636 s; p = 0.65). The fold change in tissue factor-exposing microparticles in BAL fluid inversely correlated with the fold changes in eosinophil cationic protein and myeloperoxidase in BAL fluid after virus infection (r = -0.517 and -0.528 resp., both p = 0.01).

Rhinovirus-16 challenge led to increased plasminogen activator inhibitor type-1 levels in plasma in patients with asthma (26.0 ng/mL vs. 11.5 ng/mL in healthy controls, p = 0.04). Rhinovirus-16 load in BAL showed a linear correlation with the fold change in endogenous thrombin potential, plasmin-antiplasmin complexes and plasminogen activator inhibitor type-1.

**Conclusions:**

Experimental rhinovirus infection induces procoagulant changes in the airways of patients with asthma through increased activity of tissue factor-exposing microparticles. These microparticle-associated procoagulant changes are associated with both neutrophilic and eosinophilic inflammation. Systemic activation of haemostasis increases with Rhinoviral load.

**Trial registration:**

This trial was registered at the Dutch trial registry (http://www.trialregister.nl): NTR1677.

## Introduction

It is increasingly recognized that inflammation and haemostasis are interdependent and closely linked processes that can stimulate each other [1,2]. Many chronic inflammatory diseases, including inflammatory bowel diseases [3,4], rheumatic arthritis [5,6], COPD [7-9], and sarcoidosis [10], are associated with increased coagulability of blood. This procoagulant state has also been observed in the airways of patients with stable asthma as reflected by increased levels of tissue factor (TF), thrombin-antithrombin complexes (TATc) and the plasminogen activator inhibitor-1 (PAI-1), as well as decreased levels of the natural anticoagulant protein C [11]. Local activation of coagulation may be clinically relevant for asthmatic individuals, because we have recently shown that the risk of pulmonary embolism (PE) is increased in severe asthma [12]. Recently a second study on pulmonary embolism and asthma showed not only that there is an increased risk to develop PE but that PE was associated with disease exacerbations [13].

In addition to chronic inflammatory conditions of the lung, viral and bacterial infections have been shown to induce pulmonary coagulation as well [14]. For example, elderly patients with proven respiratory viral infection had activated coagulation, as shown by increased levels of von Willebrand factor (vWF), plasmin-α2-antiplasmin complexes (PAPc), D-dimer and endogenous thrombin potential (ETP) [15]. In addition, respiratory infections were a risk factor for venous thromboembolic events in a large cohort from the general population [16].

Asthma exacerbations are most often caused by respiratory viruses [17,18], in particular rhinovirus [19]. Whether rhinovirus induced airway inflammation synergistically affects the haemostatic balance in patients with asthma is as yet unknown.

We hypothesized that rhinovirus infection enhances coagulation and reduces fibrinolysis in patients with asthma to a larger extent than in healthy control subjects. Therefore, the aim of the present study was to determine the effect of experimental rhinovirus infection on coagulation (TATc, TF-activity of microparticles, D-dimer and ETP), endothelial activation (vWF) and fibrinolysis (PAPc and PAI-1) in peripheral blood and bronchoalveolar lavage (BAL) fluid of patients with mild allergic asthma and healthy control subjects. The second aim was to assess the relationship between coagulation and fibrinolytic parameters and markers of airway inflammation in BAL fluid.

## Methods

### Subjects

Patients with mild asthma had a doctor’s diagnosis of asthma and had to meet the following criteria: baseline FEV1 > 80% predicted, PC20 (methacholine) < 8.0 mg/ml, skin-prick test positive for at least one out of 12 common aeroallergens. Healthy control subjects had the following criteria: baseline FEV1 > 80% predicted, PC20 (methacholine) > 16.0 mg/ml, skin-prick test negative for 12 common aeroallergens. All volunteers were aged between 18 and 40 years, were non-smoking or had stopped smoking more than 12 months ago with ≤ 5 pack years (PY), were negative for neutralizing antibodies against rhinovirus type 16 and did not have concomitant disease or (chronic) inflammatory condition that would interfere with this study, according to the judgment of a pulmonary physician. Patients with asthma were not allowed to use asthma medication other than short-acting β2-agonists within 2 weeks prior to the start of this study until day 6 after rhinovirus infection. Informed consent was obtained from each individual before inclusion.

### Design and procedure

**Figure 1:**
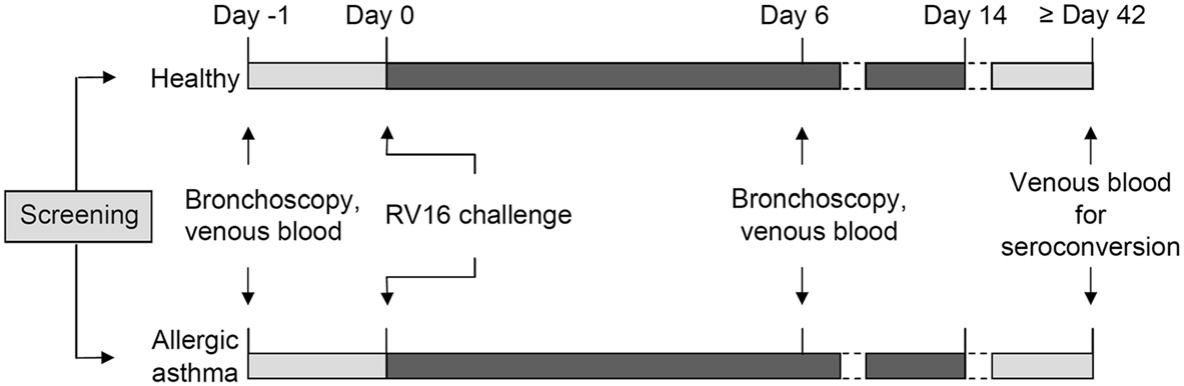
**Study design (adapted from ref. **[21]**).**

In a prospective parallel design single center study all volunteers were experimentally infected with rhinovirus type 16 (RV16) as described previously [20], but now with low-dose rhinovirus (10TCID50). In brief, upon retrieval of informed consent and subsequent screening, volunteers who met the inclusion criteria for healthy inviduals or volunteers who met the criteria for mild allergic asthma patients underwent a bronchoscopy for baseline measurements. One day later, volunteers were experimentally infected with RV16, which has been shown to cause mild common-cold symptoms in both healthy individuals and stable allergic asthma patients and to evoke a transient exacerbation of asthma symptoms. All volunteers were requested to report common cold and asthma symptoms daily until day 14 after rhinovirus challenge [20]. Diaries were collected at the final visit. Venous plasma and BAL fluid were obtained the day before and 6 days after infection. Before bronchoscopy all volunteers had used short-acting β2-agonists. Nasal swabs, brushes and BAL fluid to determine rhinovirus infection by PCR were obtained the day before and 6 days after infection. Venous blood to check for seroconversion was drawn at day 42. See Figure 1 for a flow chart of the study. More details are provided in the Additional files 1.

The study was approved by Medical Ethics Committee of the Academic Medical Centre in Amsterdam, The Netherlands. The study was registered at The Netherlands Trial Register (no. 1677). Written informed consent was obtained from all volunteers.

The primary objective of the study was to evaluate tryptophan catabolism in patients with allergic asthma and healthy subjects and the change in this metabolism after experimental rhinovirus infection [21]. Coagulation was defined as a secondary endpoint for this study. Therefore dedicated frozen citrate plasma and BAL fluid samples from patients with positive PCR or seroconversion for RV-16 after infection were analysed [21].

### Measurement of coagulation parameters

Measurements of TATc (Siemens Healthcare Diagnostics, Marburg, Germany), PAPc (DRG, Marburg, Germany) and PAI-1 (Hyphen BioMed, Andrésy, France), were performed by ELISA. D-dimer levels were determined with a particle-enhanced immunoturbidimetric assay (Innovance D-Dimer, Siemens Healthcare Diagnostics, Marburg, Germany). vWF was determined by ELISA with a polyclonal rabbit anti-human vWF antibody (A0082) as catching antibody and a horse radish peroxidase-labeled rabbit anti-human vWF antibody (P0226) as detecting antibody (both from DAKO, Glostrup, Denmark).

The presence of coagulant TF-exposing microparticles in BAL fluid was measured by a fibrin generation test (FGT) in autologous vesicle-depleted pool plasma as described before [22]. In brief, this assay determines the intrinsic capacity of the procoagualant TF-exposing microparticles in any fluid. Microparticles are isolated from the fluid by ultracentrifuge. After isolation the microparticles are added to autologous vesicle-depleted pool plasma to initiate fibrin formation. The time to clot formation decreases with increased procoagulant activity of the TF-exposing microparticles in the investigated fluid. The procoagulant activity of microparticles (time to clot formation) was measured by a Spectramax microplate reader [22]. ETP was assayed using the Calibrated Automated Thrombogram®. This assay determines the generation of thrombin in clotting plasma using a microtiter plate reading fluorometer (Fluoroskan Ascent, ThermoLab systems, Helsinki, Finland) and Thrombinoscope® software (Thrombinoscope BV, Maastricht, The Netherlands). The assay was carried out as described by Hemker et al. [23] and the Thrombinoscope® manual. More details are provided in the Additional file 1.

### Measurement of inflammatory parameters in BAL fluid

Leukocytes in BAL fluid were counted and differentiated on Quick Diff stained cytospin preparations. IL-8 was determined by Luminex according to the manufacturer's protocol (single-plex IL-8 antibody and reagent kit from Bio-Rad Laboratories, Veenendaal, The Netherlands). Myeloperoxidase (MPO) was measured by ELISA (Costar, EIA/RIA high-binding plate) using a rabbit anti-MPO (Dako A0398) as capture antibody and the biotinylated antibody as detection antibody with a well-characterised sample as MPO standard. Eosinophil cationic protein (ECP) was measured by ELISA (Nunc, Maxisorp plate) using a monoclonal mouse-anti-human ECP capture antibody (clone 614, Diagnostics Development, Uppsala Sweden), ECP standard (ImmunoCAP ECP Calibrator. Phadia, Nieuwegein, The Netherlands) and a biotinylated polyclonal rabbit-anti-human ECP detection antibody (batch ECP03-091; Diagnostics Development, Uppsala, Sweden). Both ELISAs were developed using streptavidin poly-HRP (M2051, Sanquin, Amsterdam, The Netherlands) and tetramethyl-benzidine (TMB, Merck, Darmstadt, Germany).

### Statistical analysis

Coagulation was defined as a secondary endpoint. These endpoints were defined as the changes from baseline in TATc, vWF, D-dimer, PAPc, PAI-1, and ETP in plasma and changes from baseline in TATc, vWF, FGT, D-dimer, PAPc, and PAI-1 in BAL fluid. Changes from baseline were calculated by calculating the fold change, which is the ratio of pre- and post-interventional scores (for which the following formula was used: post-interventional score/ pre-interventional score). Comparisons between both time points for each group were calculated by Wilcoxon signed rank test. Fold changes between both groups were analyzed by Mann–Whitney U test. Spearman rho correlations were used to determine associations between the fold changes of coagulation and inflammatory markers in BAL fluid. Linear correlations were calculated for the fold changes of coagulation in plasma and BAL fluid and the viral load detected in BAL-fluid. Differences were considered significant for all statistical tests at p-values less than 0.05. All reported p-values are two-sided. Analyses were performed with SPSS 18.0 (Chicago, IL, USA).

## Results

In total 28 volunteers (14 patients with mild asthma and 14 healthy controls) were included in the study. Out of the 28 patients infected with RV16, 13 patients with mild asthma and 11 healthy control subjects had a positive PCR at day 6 (either in BAL fluid or in nasal swabs or both) and/or a serologic conversion for RV16 at day 42. Two BAL fluid samples were excluded from the analysis as these samples contained >15% bronchial and squamous epithelial cells. Therefore disruption of the epithelial barrier and subsequent contamination of BAL fluid with plasma cannot be excluded for these samples. Clinical and virologic characteristics of patients and controls are shown in Table 1.

**Table 1:** Demographic data of participants*

	**Healthy controls n = 11**	**Mild asthma n = 13**	**p-value**
Female, n (%)	10 (91)	6 (46)	0.02
Age, years (range)	21 (19–31)	22 (19–29)	NS
Oral anticonceptive use, n (%)	4 (36)	3 (23)	NS
PC20 (mg/ml), geometric mean (95% CI)	>16	2.41 (1.29-4.48)	<0.001
FENO (ppb), mean (SD)	23.3 (12.9)	70.0 (36.8)	<0.05
FEV1 % pred. (range)	102 (90–121)	101 (80–123)	NS
pbFEV1, % pred. (range)	107 (98–127)	104 (83–129)	NS
PCR-positive for RV16 on day 6, n (%)	9 (81)	11 (85)	NS
PCR-positive for RV16 on day 6 in BALf, n (%)	3 (27)	7 (54)	NS
RV16 neutralization on day 42, n (%)	9 (81)	11 (85)	NS

*see Additional file 1 for inclusion and exclusion criteria.

BALf, Bronchoalveolar lavage fluid; FEV1, Forced expiratory volume in 1 sec; FENO, Fraction of exhaled Nitric oxide; PC20, provocative concentration of inhaled methacholine chloride to cause a 20 % falling in FEV1; RV16, Rhinovirus serotype 16; pb, post bronchodilator.

### Evaluation of haemostasis and fibrinolysis

#### Venous plasma

**Figure 2:**
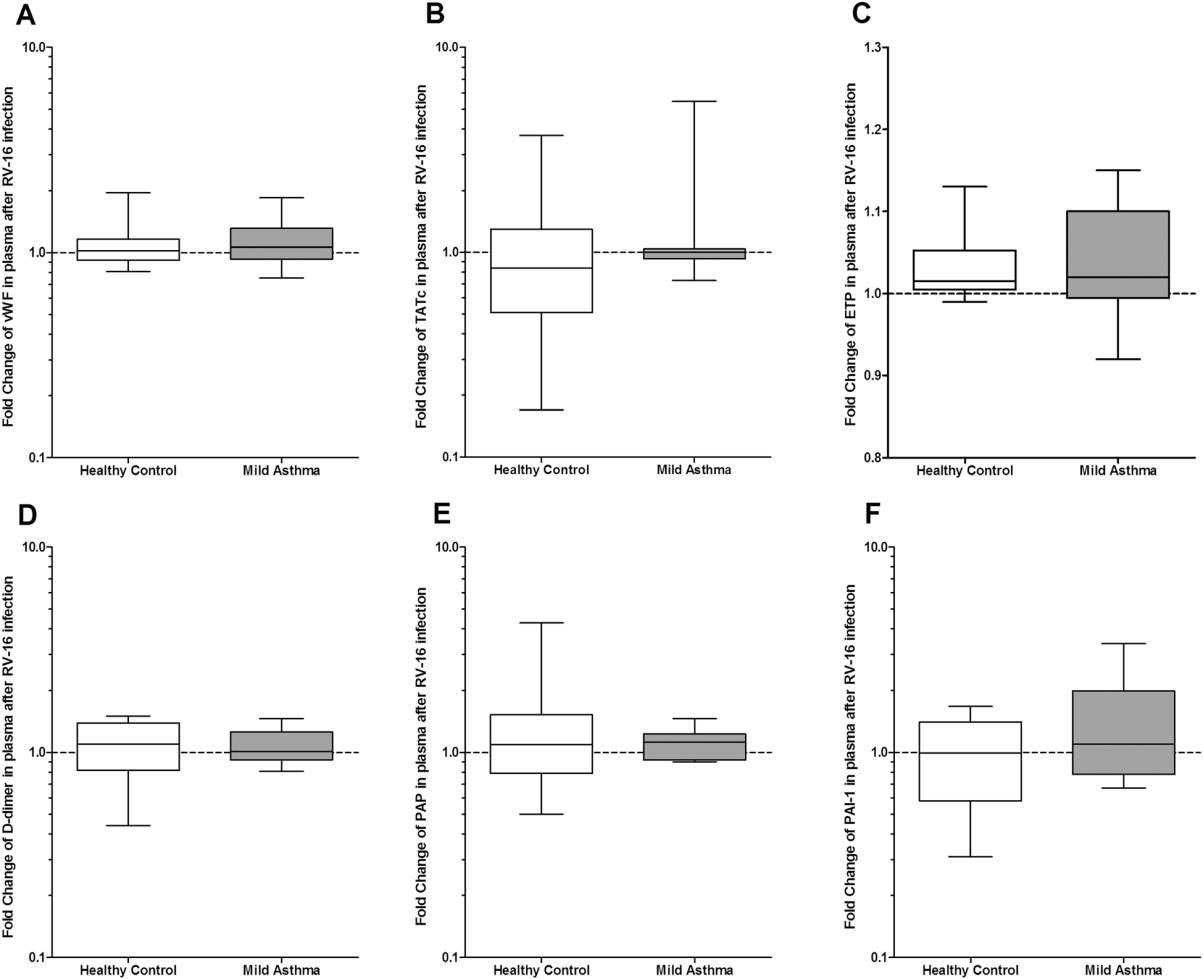
**Fold changes in haemostatic proteins and endogenous thrombin test in venous plasma after RV16-infection.** Plasma levels in healthy (white) and asthmatic (grey) individuals. of **A**: vWF, **B**: TATc, **C**: F: ETP, **D**: D-dimer, **E**: PAP-complexes, **F**: PAI-1. Box and Whisker diagram with medians and interquartile ranges.

Measurements of haemostatic and fibrinolytic markers in venous plasma at baseline and after viral infection are shown in Table 2. Of the procoagulant markers TATc was significantly higher in healthy control subjects as compared to patients with mild asthma at baseline (p = 0.02) as well as after viral infection (p = 0.03), whereas D-dimer, ETP and the marker of endothelial activation, vWF, did not differ at both time points. RV16 did not induce procoagulant changes in plasma as measured by the calculated fold changes (Figure 2A–D).

**Table 2:** Haemostatic proteins in plasma

	**Median levels of haemostatic proteins in plasma**					
	**Baseline**			**After viral infection**		
	**Control n = 11**	**Asthma n = 13**	***p-value between groups at baseline***	**Control n = 11**	**Asthma n = 13**	***p-value between groups after viral infection***
**Markers of coagulation**						
TATc (ng/mL)	2.4 (1.5–4.0)	1.5 (1.4–1.5)	*0.02*	1.95 (1.6–2.3)	1.4 (1.3–1.5)	*0.03*
vWF (% of pooled plasma)	77.0 (60.0–89.0)	99.0 (84.0–127.0)	*0.07*	87.0 (65.0–148.0)	127.0 (87.0–148.0)	*0.19*
D-dimer (ng/mL)	221 (112–422)	146 (127–173)	*0.39*	143 (113–317)	163 (144–230)	*1.00*
ETP (nM.min)	1653 (1340–1663)	1446 (1290–1644)	*0.25*	1660 (1351–1743)	1494 (1321–1645)	*0.19*
**Markers of fibrinolysis**						
PAP (ng/mL)	385 (261–618)	327 (276–391)	*0.17*	534 (311–786)	320 (303–467)	*0.047*
PAI-1 (ng/mL)	13.0 (11.0–21.0)	16.0 (12.0–24.0)	*0.28*	11.5 (9.0–16.0)	26.0 (15.0–33.0)*	*0.04*

Numbers are medians with IQR in parentheses.

von Willebrand factor is expressed as percentage of normal pool plasma.

*p < 0,05 versus baseline.

The fibrinolytic marker PAP and PAI-1 did not differ between both groups at baseline. Although the fold changes of the fibrinolytic parameters did not change significantly (Figure 2E and F), PAP levels were significantly lower (p = 0.047) and PAI-1 levels significantly higher (p = 0.04) after RV16 infection in asthma patients than in healthy individuals.

#### BAL-fluid

**Figure 3:**
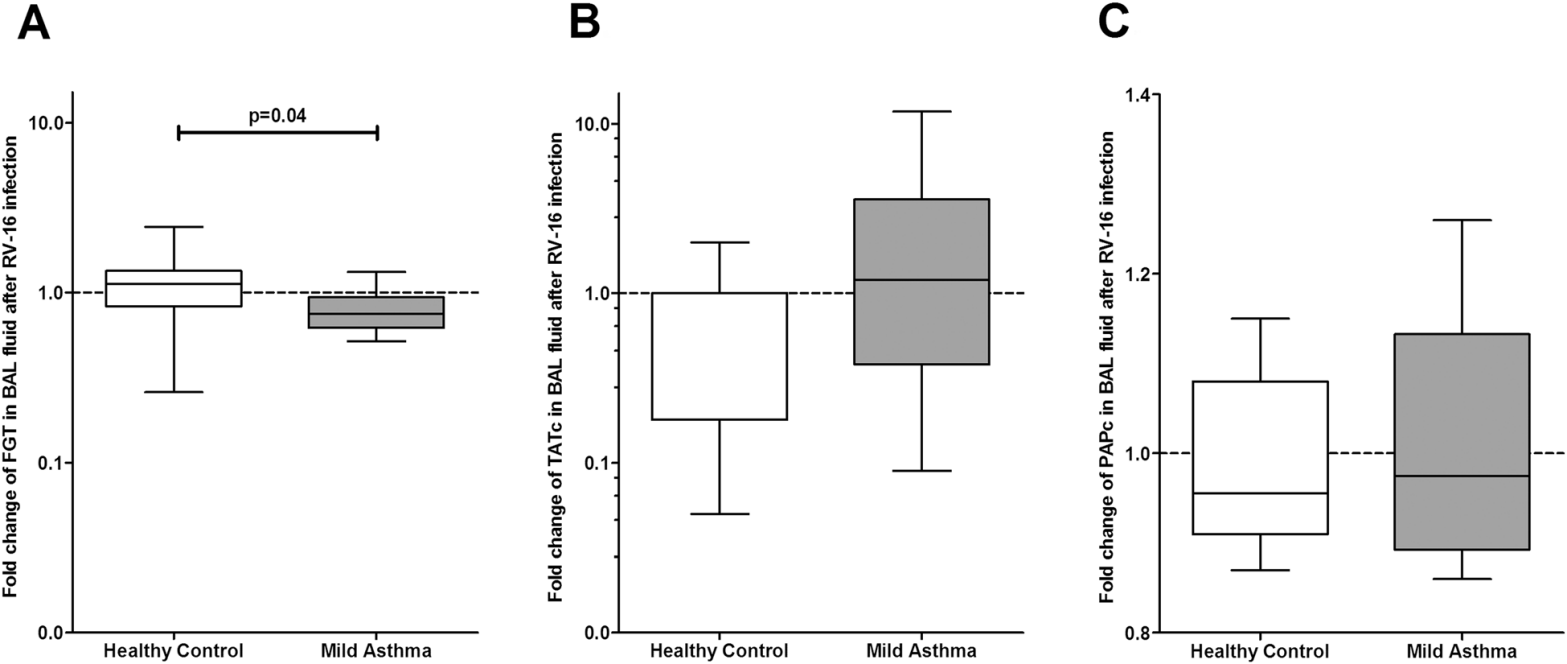
**Fold changes in haemostatic proteins and fibrin generation test in BAL-fluid after RV16-infection.** BALF levels in healthy (white) and asthmatic (grey) individuals of **A**: FGT, **B**: TATc, **C**: PAPc. Box and Whisker diagram with medians and interquartile ranges.

Amongst the procoagulant markers (TATc, D-dimer), the endogenous capacity of the TF-exposing microvesicles (FGT) and the marker of endothelial activation (vWF) only TATc and FGT were detectable in BAL-fluid (Table 3). TATc levels in BAL-fluid were not different between both groups at baseline and after RV16 challenge nor did the fold change from baseline differ between both groups. However, FGT being similar in both groups at baseline, shortened in patients with asthma after viral infection (t = -1: 706 s vs. t = 6: 498 s; p = 0.02), but not in healthy control subjects (t = -1: 693 s vs. t = 6: 636 s; p = 0.65; Table 3) Change from baseline of FGT after RV16 challenge did significantly differ between patients with mild asthma and healthy control subjects (p = 0.04; Figure 3A and B).

**Table 3:** Haemostatic proteins and fibrin generation test in BAL-fluid

	**Median levels of haemostatic proteins in BAL-fluid**					
	**Baseline**			**After viral infection**		
	**Control n = 10**	**Asthma n = 13**	***p-value between groups at baseline***	**Control n = 11**	**Asthma n = 12**	***p-value between groups after viral infection***
FGT (s)	693 (486–853)	706 (513–855)	*0.88*	636 (541–859)	498 (399–596)*	*0.053*
TATc (ng/mL)	0.70 (0.25-4.43)	0.80 (0.50-2.10)	*0.85*	0.50 (0.25-0.60)	0.75 (0.53-2.73)	*0.058*
PAP (ng/mL)	34.5 (30.0-53.0)	44.0 (37.0-45.5)	*0.15*	34.0 (30.0-55.0)	42.5 (38.3-47.0)	*0.14*

Numbers are medians with IQR in parentheses.

*p < 0,05 versus baseline.

Of the fibrinolytic markers, only PAPc was detectable in BAL-fluid. PAPc levels did not differ between both time points nor did the fold change from baseline differ between both groups.

### Associations between coagulation and inflammatory parameters

As compared to healthy control subjects, patients with mild asthma had significantly higher levels of eosinophils (Median 1.0% vs. 0.25%, resp. p < 0,01) and ECP (Median 286 pg/ml vs. 159 pg/ml, resp. p < 0,05) at both timepoints. Asthmatic individuals showed an increase in ECP (Median 286 pg/ml to 507 pg/ml after viral infection, p = 0,04) and IL-8 (Median 0.34 pg/ml to 1.42 pg/ml after viral infection, p = 0,04) in BALF after rhinovirus exposure [21]. However the fold changes from baseline after infection did not change significantly between both groups (ECP p = 0,10 and for IL-8 p = 0,10). Spearman correlations were calculated to explore possible associations between haemostatic and inflammatory markers. At baseline, both FGT and TATc showed a weak correlation with IL-8 (R_IL-8_ = -0.455 for FGT (p = 0.03) and R_IL-8_ = 0.461 for TATc, both p = 0.02). No correlations were found for ECP nor for MPO. After viral infection, the fold changes of FGT and TATc in BAL-fluid showed a significant correlation with ECP and MPO, while TATc also correlated with IL-8 in BAL-fluid (Table 4). Figures of these associations are shown in the Additional file 2.

**Table 4:** Correlations between fold changes of inflammatory and haemostatic parameters in BAL-fluid after viral infection

**Spearman’s correlation coefficient**			
	**Fold changes**		
	**FGT**	**TATc**	**PAP**
Eosinophils	-0.103	0.363	0.190
ECP	-0.517*	0.465*	0.233
Neutrophils	-0.434*	0.632**	0.217
MPO	-0.528*	0.806***	0.370
IL-8	-0.413	0.579**	-0.006

Significance level: *< 0.05; **< 0.01; ***< 0.001.

MPO myeloperoxidase; ECP eosinophili cationic protein; FGT fibrin generation test; TATc thrombin-antithrombin complex; PAP plasmin-antiplasmin complex.

### Associations between RV-load in BAL fluid and haemostatic proteins in plasma and BAL fluid

**Figure 4:**
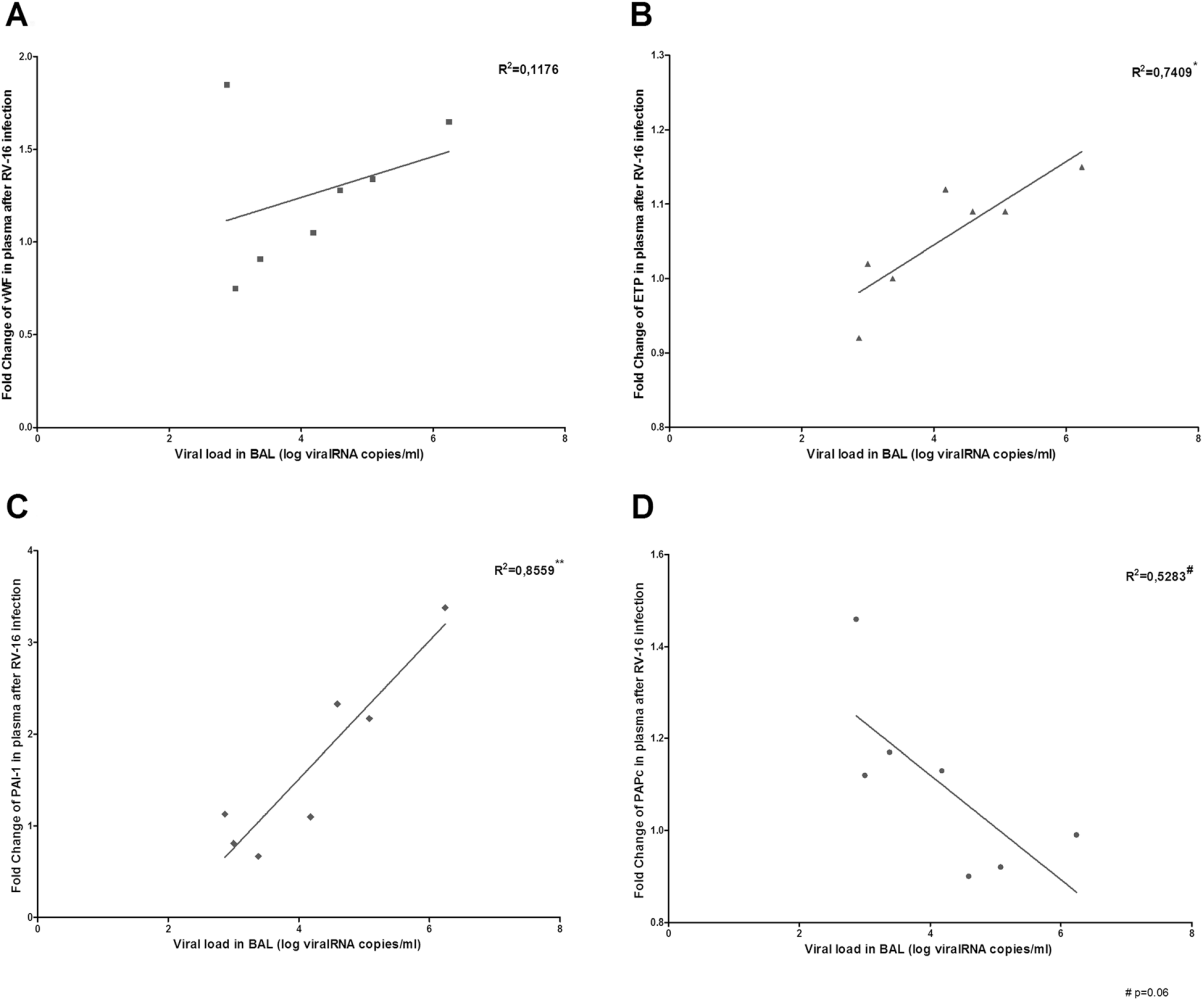
**Fold changes in haemostatic proteins and endogenous thrombin test in venous plasma versus RV-16 load in BAL fluid. A**: vWF, **B**: ETP, **C**: PAI-1 and **D**: PAPc. Scatter-dot diagram with interpolation line. (*p < 0.05, **p < 0.01).

RV-16 was detected in BAL fluid in 7 asthmatic patients and 3 healthy controls. Only the mild asthmatic patients were analysed as the group of healthy controls was too small. ETP showed a significant linear correlation with RV16-load in BAL fluid (Figure 4, R^2^ = 0.7409 p = 0.01). PAI-1 also showed a significant positive linear correlation with RV16-load (R^2^ = 0.8559, p = 0.003), while an inverse linear correlation with PAPc was found (R^2^ = 0.5283, p = 0.06). vWF did not show a significant correlation, because of a single outlier (Figure 4A, R^2^ = 0,1176, p = 0,46) When the outlier was omitted from the analyses, the relationship was very significant (R^2^ = 0.982, p = 0,0001). The fold change in TATc and D-dimer did not correlate in these patients with the RV16-load in BAL fluid, nor did the haemostatic markers in BAL fluid correlate with the viral load in BAL fluid.

## Discussion

This study shows that rhinovirus infection in patients with mild asthma, but not in healthy subjects, increases procoagulant activity in the pulmonary environment as evidenced by increased microparticle-associated TF activity (FGT) in BAL fluid. Moreover, fold changes in eosinophil cationic protein and myeloperoxidase were both associated with the fold changes in FGT and TATc levels in BAL fluid after viral infection. This suggests that rhinovirus infection increases the local procoagulant activity in the airways through both eosinophilic and neutrophilic airway inflammation. In addition to local procoagulant activity, RV16-load showed linear correlations with markers of fibrinolysis and haemostatic activity in plasma.

This is the first study investigating the effect of experimental virus infection on activation of coagulation in patients with asthma. We observed an increase in the intrinsic capability to coagulate in BAL fluid, which is associated with eosinophilic and neutrophilic inflammation. This confirms previous studies showing that inflammatory and allergic asthma exacerbations induce coagulation in the airways as reflected by increased levels of TATc, TF activity and factor Xa [24-34]. Both fibrin and thrombin have been implicated in airway hyperresponsiveness and allergic airway inflammation [35], which may be the result of inactivation of surfactant and/or the formation of sputumplugs [36].

The increase in procoagulant activity in patients with asthma after rhinovirus infection can be explained by several mechanisms. Firstly, coagulant TF-exposing microparticles are likely to play a key role in the local activation of coagulation in the airways of asthmatic individuals, since these microparticles have been shown to be indispensable to initiate coagulation through the extrinsic pathway [22]. Besides activation of coagulation, microparticles have been shown to be mediators of inflammation during infection [6,37].

TF-exposing microparticles derived from eosinophils and neutrophils have been described in related eosinophilic diseases, like Churg Strauss vasculitis and bullous pemphigoid [38-40]. However, eosinophils as the potential source of coagulant TF-exposing microparticles remain controversial, since TF mRNA is below the detection limit in both resting and stimulated eosinophils [41,42]. Monocytes may be able to transfer TF to microparticles originating from eosinophils and neutrophils. This may explain the associations we observed in BAL-fluid between FGT and TATc with ECP and MPO and fits with the observation by Bouwman et al. [25] that virus-infected monocytes show a higher TF expression.

This study extends the role of increased procoagulant activity as described in previous studies to infectious aetiologies of asthma exacerbation [31,32]. For example, we observed a decreased fibrinolysis in plasma of patients with mild asthma after RV16 infection, while markers of fibrinolysis were undetectable in BAL fluid. In addition, we observed linear correlations between RV16-load in BAL-fluid and systemic PAI-1 and ETP and an inverse correlation with systemic PAPc were found. Two possible explanations could be provided for these differences in plasma. First, IFN-γ treatment has been shown to decrease levels of PAI-1 in healthy controls [43,44]. Since interferon production in patients with asthma has been shown to be impaired [45-47] this might explain the increased levels of PAI-1 in the present study. Although systemic fibrinolysis is impaired in patients with mild asthma, TATc levels in plasma were higher in healthy control subjects, while the systemic fold changes from baseline in TATc, ETP, vWF and PAI-1 are increased in the mild asthmatic group. A possible explanation may be that there is no suitable trigger at baseline leading to decreased TATc levels in plasma of patients with mild allergic asthma. Whether there are compensatory mechanisms leading to lower TATc levels in plasma of asthmatic individuals as compared to healthy individuals is as yet unknown and may be subject for further research. It is important to note that after infection with a mild rhinovirus infection the changes from baseline in plasma are larger in the mild asthma group than in the healthy group and illustrates the increased capability for coagulation.

Secondly, the linear correlations between RV16-load in BAL-fluid and systemic PAI-1 and ETP and inverse correlation with systemic PAPc observed for patients with mild asthma point towards endothelial activation by RV16. vWF and PAI-1 are stored in endothelial cells [48]. Quiescent endothelial cells are generally driving the haemostatic balance in favour of fibrinolysis through activated protein C generation and production of tissue-type plasminogen activator. Upon a stimulus, for example an infection, this balance is changed into a procoagulant state by release of amongst others vWF and expression of PAI-1 [48,49]. Our current study points towards activation of endothelial cells by rhinovirus, although it is unclear whether the activation is directly or indirectly. Endothelial cells express intercellular adhesion molecule 1 (ICAM-1), which is not only an important molecule for leukocyte adhesion [48], it is also the receptor for rhinovirus to infect human cells [50]. However, the linear correlation between rhinovirus in the airway lumen and procoagulant activity in plasma points towards indirect activation of endothelial cells by rhinovirus, for example through an inflammatory mediators produced by airway epithelial cells [50]. Either way, activation of the endothelium results in an increase of PAI-1 and vWF. The increase in PAI-1 may explain the decrease in PAPc [51], while the increase in ETP might be explained by the increased levels of factor VIII [3,52] commonly associated with an increase in vWF and other pro-coagulants [53]. Both impaired IFN production and endothelial activation may play a role in the increased systemic haemostatic activity found in patients with mild asthma. Our results are in line with previous studies about the activation of haemostasis in healthy controls *in vitro* (viral infection of endothelial cells and monocytes) [54,55] and *in vivo* (elderly patients with acute respiratory tract infections) [56].

The strength of our study is the experimental design, which provided standardized exposure and comparable time points of post-infection sampling. In addition, the patients were free of steroids, which may affect inflammatory and coagulant outcomes. However, we cannot exclude the influence of potential confounding factors in the present outcomes. First, the male/female ratio was different between the two groups, with more female subjects in the control group than in the group of patients with asthma. Since many women used oral contraceptives, this might have influenced coagulation activity [57,58]. However, we do not think that this factor biased our results since oral contraceptive users were equally distributed between the two groups and fibrinolysis was only impaired in patients with asthma.

Secondly, we did not observe significant changes for some relevant coagulation parameters in BAL fluid and plasma. This might relate to the mild nature of the experimental rhinovirus infection (i.e. low infectious dose and mild rhinovirus strain). We used this method to infect volunteers because it most closely resembles the natural course of rhinovirus infection. In addition, the procedure has been proven to be safe in patients with mild asthma and resulted in increase in common cold symptoms in both asthmatic and healthy individuals. The effects on coagulation are likely more pronounced after a rhinovirus-induced exacerbation in patients with moderate to severe asthma or in more severe disease exacerbations (i.e. influenza).

Another explanation for the non significant changes of coagulant parameters in BAL fluid and plasma could be the use of short-acting beta-2-agonists. Although it is suggested that beta-2 agonists may have a small anti-inflammatory effect [59,60], we believe this has not influenced our results as both groups of volunteers used salbutamol during the spirometry test just before the bronchoscopy with BAL.

Thirdly, the study was limited by the fact that some haemostatic parameters were below the detection limit in BAL fluid which could be explained by the timing of the second bronchoscopy and/or the dilution involved with bronchoalveolar lavage. This is a common problem often encountered and difficult to circumvent in this type of studies due to the dilution involved with bronchoalveolar lavage. Still, our data provides evidence of increased procoagulant activity in the airways of patients with asthma after viral infection, which correlates with inflammation, and thus matches with our hypothesis.

And finally, one could argue that a larger study population would have shown more procoagulant activity and systemic procoagulant changes. Based on our present findings, we calculated that we would need more than 1000 volunteers to reach statistical significance, which is neither ethical nor feasible. Evaluation of coagulation after rhinovirus infection in patients with more severe asthma would be more relevant as these patients are more at risk for venous thromboembolism [12].

Although this study is observational in nature, the findings of the present study may have important clinical implications. Patients with asthma have an increased risk of pulmonary embolism [12,13] which is associated with asthma exacerbations [13]. Our results indicate for the first time that even a mild rhinovirus infection in patients with mild asthma induces procoagulant activity in the airways and a possible systemic increase in haemostatic activity. One could hypothesize that patients with severe asthma have more procoagulant activity and could therefore be at increased risk to develop acute thromboembolic events due to rhinovirus infections. In this perspective, it would be required to further investigate a potential causal relationship between coagulation and eosinophilic inflammation in asthmatic airways and to further explore the nature of TF-bearing microparticles in patients with asthma. Furthermore, follow-up studies should focus on activation of haemostasis in patients with moderate to severe asthma as well as procoagulant changes induced by influenza and respiratory syncytial virus (RSV), as both influenza and RSV are frequently isolated in patients that are hospitalized for their asthma exacerbation [18].

## Conclusions

In conclusion we have provided evidence of local procoagulant activity after rhinovirus infection in the airways of patients with asthma, which is associated with the intensity of eosinophilic and neutrophilic airway inflammation. As rhinovirus infection is a very common trigger of asthma exacerbations the clinical consequences of this induction of procoagulant activity deserve further investigation, in particular in patients with severe asthma who have already an increased risk of myocardial infarction, stroke and venous thromboembolism [12,61].

## Abbreviations

BAL: Bronchoalveolar lavage; COPD: Chronic obstructive pulmonary disease; DD: Doubling doses of methacholine; ECP: Eosinophil cationic protein; ELISA: Enzyme-linkes immunosorbant assay; ETP: Endogenous thrombin potential; FENO: Fraction of exhaled Nitric oxide; FEV1: Forced expiratory volume in 1 second; FGT: Fibrin generation test; ICAM-1: Intercellular adhesion molecule 1; IFN-γ: Interferon gamma; IL-8: Interleukin 8; IQR: Interquartal range; mRNA: Messenger ribosomal nucleic acid; MPO: Myeloperoxidase; pb: Post bronchodilator; PAI-1: Plasminogen activator inhibitor-1; PAPc: Plasmin-α2-antiplasmin complexes; PC20: Provocative concentration of inhaled methacholine to cause a 20% falling in FEV1; PCR: Polymerase chain reaction; PE: Pulmonary embolism; PY: Packyears; RSV: Respiratory syncytial virus; RV16: Rhinovirus type 16; TATc: Thrombin-antithrombin complexes; TF: Tissue factor; vWF: von Willebrand factor.

## Competing interests

The authors declare that they have no competing interests.

## Authors’ contributions

Conception and design: KS, RL, PS, CJM, PWK, and EHDB. Data collection: KS, MP CJM Analysis and interpretation: CJM, PWK, JCMM, RM, KCW, TP, RN, EHDB, SLJ, KS, Drafting and editing manuscript: all authors. All authors read and approved the final manuscript.

## Supplementary Material

Additional file 1 Evaluation of coagulation activation after Rhinovirus infection in patients with asthma and healthy control subjects: an observational study. Click here for file

Additional file 2: Figure E1 Associations between fold changes of inflammatory parameters and hemostatic proteins and fibrin generation test in BAL-fluid after RV16-infection. Click here for file
