# Supplementary material for: Evaluation of coagulation activation after Rhinovirus infection in patients with asthma and healthy control subjects: an observational study
Source: Respir Res. 2014 Feb 7;15(1):14. doi: 10.1186/1465-9921-15-14 (PMC3922343; doi:10.1186/1465-9921-15-14)
Supplement: Additional file 1 — Evaluation of coagulation activation after Rhinovirus infection in patients with asthma and healthy control subjects: an observational study. [file 1465-9921-15-14-S1.doc]

# **Online supplement for:**

Evaluation of coagulation activation after Rhinovirus infection in patients with asthma and healthy control subjects: an observational study

C.J. Majoor^1^, M.A. van de Pol^2^, P.W. Kamphuisen^8^, J.C.M. Meijers^3^, Richard Molenkamp^4^, Katja C. Wolthers^4^, T. van der Poll^5^, R. Nieuwland^6^, S.L. Johnston^9^, P.J. Sterk^1^ , E.H.D. Bel^1^, R. Lutter^2^, K. van der Sluijs^1,2,7^

Dept. of ^1^Respiratory Medicine, ^2^Experimenal Immunology, ^3^Experimental Vascular Medicine, ^4^Department of Medical Microbiology, ^5^Center of Experimental and Molecular Medicine, ^6^Clinical Chemistry, and ^7^Laboratory of Experimental Intensive Care and Anesthesiology, Academic Medical Centre, Meibergdreef 9, 1105 AZ, Amsterdam, The Netherlands

^8^Dep. of Vascular Medicine, University Medical Centre Groningen, Hanzeplein 1, 9700 HB, Groningen, The Netherlands;

^9^Dep. of Respiratory Medicine, National Heart and Lung Institute, Imperial College London, Norfolk Place, London W2 1PG, United Kingdom

Corresponding author:

C.J. Majoor

Dept. of Respiratory Medicine

Academic Medical Center

P.O. Box 22660

1100 DE Amsterdam

The Netherlands

Telephone: +31-20-5664356

Fax number: +31-20-5669001

Email: [c.j.majoor@amc.uva.nl](mailto:c.j.majoor@amc.uva.nl)

# **I. Methods**

# **Inclusion and exclusion criteria**

Inclusion criteria:

General:

- Aged between 18-40 years
- Non-smoking or stopped smoking more than 12 months ago and 5 pack years or less
- Able to give written and dated informed consent prior to any study-specific procedures

Asthmatic patients:

- Baseline bronchial hyperresponsiveness to methacholine PC20 < 8.0 mg/
- Skin-prick test positive to common allergens
- Clinically stable, for patients with mild asthma this means no exacerbations within the last 6 months prior to the study, as indicated by a course of systemic steroids or antibiotics.
- FEV1 at baseline at least 70% of the predicted value
- Patients are treated with inhaled beta-2-agonists on demand only and have not used (inhaled) steroids for at least 2 weeks prior to the study.
- No other clinically significant abnormality on history and clinical examination

Healthy subjects:

- FEV1 at baseline at least 80% of the predicted value
- Baseline PC20 methacholine >16.0 mg/ml
- Skin-prick test negative to common allergens.

Exclusion criteria:

- Circulating antibodies against RV16.
- In close contact with young children (< 2 years), either professional or family related.
- Use of heparin, LMWH, NSAID or vitamin K antagonists.
- Women who are pregnant or lactating or who have a positive urine pregnancy test at screening
- Concomitant disease or condition which could interfere with the conduct of the study, or for which the treatment might interfere with the conduct of the study, or which would, in the opinion of the investigator, pose an unacceptable risk to the patient in this study
- Unwillingness or inability to comply with the study protocol for any other reason

# **Clinical procedures**

*Virus detection.*

Rhinovirus and other respiratory viruses were detected by measuring viral load and polymerase change reaction (PCR) according to previously established protocols[2]. Viral load was determined by standard cell-culture techniques (culture in LLC-MK2, Hep2 and HEL cells, also to exclude intercurrent viral infection)[3]. In addition, RNA was extracted using Trizol to determine viral load by real-time quantitative PCR[4].

*Pulmonary function test*

Spirometry and methacholine provocation test were performed according to European Respiratory Society (ERS) recommendations using a Jaeger pneumotach[5].

*Bronchoalveolar lavage and venous blood collection*

Bronchoalveolar lavage and citrate-anticoagulated blood (0.32%) were collected from the study population at day -1 and day 6. Bronchoscopy was performed by an experienced pulmonologist using a flexible fiberoptic videobronchoscope as described previously[6]. Bronchoalveolar lavage fluid (BALF) was obtained by instillation of eight aliquots of 20 ml of prewarmed 0.9% NaCl and aspirated immediately by slow suction. Peripheral venous blood was obtained with a 19 gauge needle through a Vacutainer system from the brachial vene.

BALF and blood were centrifugated at 1800G for 20 minutes at 4°C to prepare cell-free BALF and plasma. After centrifugation the BALF and plasma were immediately frozen at -80°C until use.

***Analysis of coagulation parameters***

# Analysis of Microparticles/exosomes

Microparticles and exosomes were isolated from BALF after centrifugation for 30 minutes at 18,890 *g* and 4 °C. After isolation the microparticles were added to normal pool plasma with antibodies against either human TF or coagulation factor VII(a) to inhibit the extrinsic coagulation pathway, and, as control, an antibody against human coagulation factor XII(Sanquin). The pool plasma was depleted from endogenous vesicles by ultracentrifugation (1 hour at 154,000 *g*) before use. This plasma (84 µl) was then mixed with the isolated microparticles plus either saline (3 µl) or antibody (3 µl), and incubated for 5 minutes at 37 °C in a 96 well plate. Clotting was initiated by addition of CaCl_2_ (15 µl; 0.1 M). Fibrin (clot) formation was monitored by measuring the optical density of the plasma (λ = 405 nm) on a Spectramax microplate reader (Molecular Devices Corp.; Sunnyvale, CA) at 37 °C for 1 hour. In addition, also the ability of BALF to trigger coagulation was studied directly, i.e. by addition of BALF directly to vesicle-depleted normal pool plasma in the presence or absence of before-mentioned antibodies.

Although one could argue that freezing may changes TF activity by inverting the orientation of the microparticles, our experience is that a single freeze-thaw cycle does not affect the ability of microparticle-exposed TF to initiate coagulation (R. Nieuwland, personal communication).

## Measurement of coagulation and fibrinolytic parameters

### Measurements of thrombin-antithrombin complexes (TATc) (Siemens Healthcare Diagnostics, Marburg, Germany), plasmin-alpha2-antiplasmin complexes (PAP) (DRG, Marburg, Germany), and plasminogen activator inhibitor-1 antigen (PAI-1) (Hyphen BioMed, Andrésy, France) were performed by ELISA. vWF was determined by ELISA with a polyclonal rabbit anti-human vWF antibody (A0082) as catching antibody and a horse radish peroxidase-labeled rabbit anti-human vWF antibody (P0226) as detecting antibody (both from DAKO, Glostrup, Denmark). D-dimer levels were determined with a particle-enhanced immunoturbidimetric assay (Innovance D-Dimer, Siemens Healthcare Diagnostics, Marburg, Germany).

### The Calibrated Automated Thrombogram® assays the generation of thrombin in clotting plasma using a microtiter plate reading fluorometer (Fluoroskan Ascent, ThermoLab systems, Helsinki, Finland) and Thrombinoscope® software (Thrombinoscope BV, Maastricht, The Netherlands). The assay was carried out as described by Hemker et al.[7] and the Thrombinoscope® manual. Coagulation was triggered by recalcification in the presence of 5 pM recombinant human tissue factor (Innovin®, Siemens, Marburg, Germany), 4 μM phospholipids, and 417 μM fluorogenic substrate Z-Gly-Gly-Arg-AMC (Bachem, Bubendorf, Switzerland). Fluorescence was monitored using the Fluoroskan Ascent fluorometer (ThermoLabsystems, Helsinki, Finland), and the ETP (endogenous thrombin potential, area under the curve of the thrombogram), peak thrombin and lag time were calculated using the Thrombinoscope® software (Thrombinoscope BV).

### Reference List

1. Sterk PJ, Fabbri LM, Quanjer PH, Cockcroft DW, O'Byrne PM, Anderson SD, Juniper EF, Malo JL: **Airway responsiveness. Standardized challenge testing with pharmacological, physical and sensitizing stimuli in adults. Report Working Party Standardization of Lung Function Tests, European Community for Steel and Coal. Official Statement of the European Respiratory Society.** *Eur Respir J Suppl* 1993, **16:**53-83.

2. Message SD, Laza-Stanca V, Mallia P, Parker HL, Zhu J, Kebadze T, Contoli M, Sanderson G, Kon OM, Papi A et al.: **Rhinovirus-induced lower respiratory illness is increased in asthma and related to virus load and Th1/2 cytokine and IL-10 production.** *Proc Natl Acad Sci U S A* 2008, **105:**13562-13567.

3. Grunberg K, Sharon RF, Sont JK, In 't Veen JC, Van Schadewijk WA, De Klerk EP, Dick CR, Van Krieken JH, Sterk PJ: **Rhinovirus-induced airway inflammation in asthma: effect of treatment with inhaled corticosteroids before and during experimental infection.** *Am J Respir Crit Care Med* 2001, **164:**1816-1822.

4. Contoli M, Message SD, Laza-Stanca V, Edwards MR, Wark PA, Bartlett NW, Kebadze T, Mallia P, Stanciu LA, Parker HL et al.: **Role of deficient type III interferon-lambda production in asthma exacerbations.** *Nat Med* 2006, **12:**1023-1026.

5. Miller MR, Hankinson J, Brusasco V, Burgos F, Casaburi R, Coates A, Crapo R, Enright P, van der Grinten CP, Gustafsson P et al.: **Standardisation of spirometry.** *Eur Respir J* 2005, **26:**319-338.

6. Maris NA, de Vos AF, Dessing MC, Spek CA, Lutter R, Jansen HM, van der Zee JS, Bresser P, van der Poll T: **Antiinflammatory effects of salmeterol after inhalation of lipopolysaccharide by healthy volunteers.** *Am J Respir Crit Care Med* 2005, **172:**878-884.

7. Hemker HC, Giesen P, Al Dieri R, Regnault V, de Smedt E, Wagenvoord R, Lecompte T, Beguin S: **Calibrated automated thrombin generation measurement in clotting plasma.** *Pathophysiol Haemost Thromb* 2003, **33:**4-15.

***II. Results***

Figure E1 Associations between fold changes of inflammatory parameters and hemostatic proteins and fibrin generation test in BAL-fluid after RV16-infection.
