# Supplementary figures and images for: Evaluation of coagulation activation after Rhinovirus infection in patients with asthma and healthy control subjects: an observational study
Source: Respir Res. 2014 Feb 7;15(1):14. doi: 10.1186/1465-9921-15-14 (PMC3922343; doi:10.1186/1465-9921-15-14)

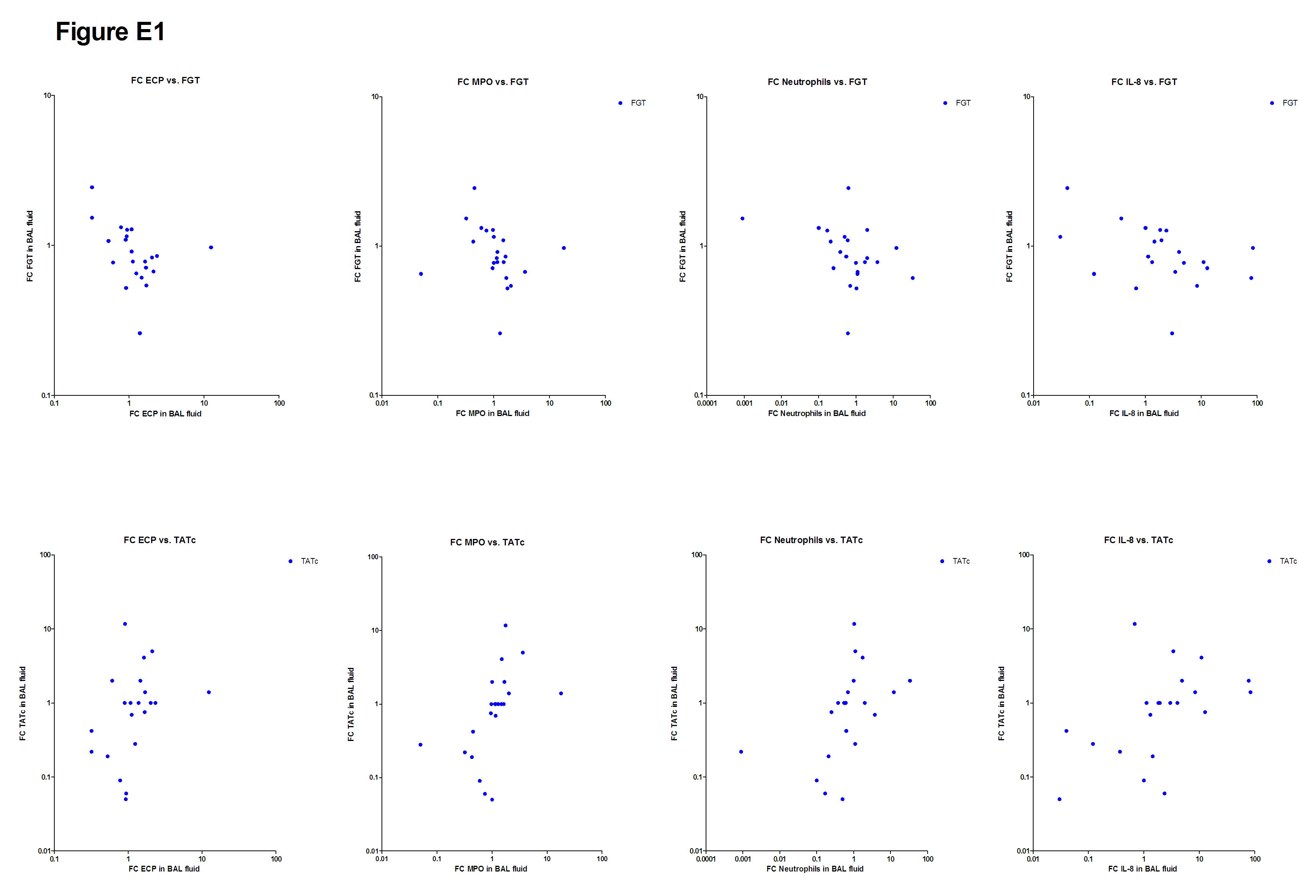

Supplement: Additional file 2: Figure E1 — Associations between fold changes of inflammatory parameters and hemostatic proteins and fibrin generation test in BAL-fluid after RV16-infection. [file 1465-9921-15-14-S2.bmp]
